# Diseases of the Joints

**Published:** 1887-05

**Authors:** 


### BOOK REVIEWS AND NOTICES.

DISEASES OF THE JOINTS. *By* HOWARD MARSH, F. R. C. S. *Duodecimo, pp. viii., 461. Sixty-four wood engravings and a chromo-lithographic plate. Philadelphia:* LEA BROTHERS & CO. *Chicago:* A. C. McCLURG & CO.

The author avows an intention to "confine himself mainly to a description of diagnosis and treatment." It is to be regretted that he has done so, for if recent pathology had been given more attention, the work would be valuable as a short text-book to under-graduates.

About two-thirds of the contents are devoted to a description of symptoms, diagnosis and treatment of joint-diseases in general; the other chapters give a review of the diseases as affecting particular joints.

The subject of traumatic dislocation is omitted entirely, and the question of tuberculosis is given only slight attention.

A number of new and valuable suggestions with regard to mechanical appliances are made.

The engravings are clear and many of them new.

References are comparatively few and confined to the works of English authors.

In its present edition the book will be found of use to the student as a short manual of diagnosis, and may give to the practitioner some new hints upon treatment.

